# Comparison of Mantoux and Tine Tuberculin Skin Tests in BCG-Vaccinated Children Investigated for Tuberculosis

**DOI:** 10.1371/journal.pone.0008085

**Published:** 2009-11-30

**Authors:** Wenli Pan, Lyness Matizirofa, Lesley Workman, Tony Hawkridge, Willem Hanekom, Hassan Mahomed, Gregory Hussey, Mark Hatherill

**Affiliations:** 1 Department of Health, Haikou, Hainan Province, China; 2 German Academic Exchange Service (DAAD) Program, Charite Medical University, Berlin, Germany; 3 South African Tuberculosis Vaccine Initiative, Institute of Infectious Diseases & Molecular Medicine, University of Cape Town, Cape Town, South Africa; 4 School of Child & Adolescent Health, University of Cape Town, Cape Town, South Africa; 5 Aeras Global TB Vaccine Foundation, Rockville, Maryland, United States of America; McGill University, Canada

## Abstract

**Background:**

Tuberculin skin tests (TSTs) are long-established screening methods for tuberculosis (TB). We aimed to compare agreement between the intradermal Mantoux and multipuncture percutaneous Tine methods and to quantify risk factors for a positive test result.

**Methodology/Principal Findings:**

1512 South African children younger than 5 years of age who were investigated for tuberculosis (TB) during a Bacille Calmette Guerin (BCG) trial were included in this analysis. Children underwent both Mantoux and Tine tests. A positive test was defined as Mantoux ≥15 mm or Tine ≥ Grade 3 for the binary comparison. Agreement was evaluated using kappa (binary) and weighted kappa (hierarchical). Multivariate regression models identified independent risk factors for TST positivity. The Mantoux test was positive in 430 children (28.4%) and the Tine test in 496 children (32.8%, p<0.0001), with observed binary agreement 87.3% (kappa 0.70) and hierarchical agreement 85.0% (weighted kappa 0.66). Among 173 children culture-positive for *Mycobacterium tuberculosis*, Mantoux was positive in 49.1% and Tine in 54.9%, p<0.0001 (kappa 0.70). Evidence of digit preference was noted for Mantoux readings at 5 mm threshold intervals. After adjustment for confounders, a positive culture, suggestive chest radiograph, and proximity of TB contact were risk factors for a positive test using both TST methods. There were no independent associations between ethnicity, gender, age, or over-crowding, and TST result.

**Conclusions/Significance:**

The Tine test demonstrated a higher positive test rate than the Mantoux, with substantial agreement between TST methods among young BCG-vaccinated children. TB disease and exposure factors, but not demographic variables, were independent risk factors for a positive result using either test method. These findings suggest that the Tine might be a useful screening tool for childhood TB in resource-limited countries.

## Introduction

Tuberculin skin tests (TSTs) are long-established screening methods for tuberculosis (TB) infection that detect the cell-mediated response to inoculation of a mixture of *Mycobacterium tuberculosis* antigens, some of which are common to bacille Calmette-Guerin (BCG) and non-tuberculous mycobacteria (NTM)[1], [2]. Traditionally, TSTs have also formed part of the decision-making pathway for the diagnosis of childhood TB disease[3]. In recent years, there has been intense research interest in diagnosis of latent TB infection by quantitative interferon-gamma release assays (IGRAs), which may offer rapid turnaround and greater specificity[4]. Authorities in some developed countries have recommended that the TST be replaced completely by the IGRA, although the evidence supporting the use of IGRAs for diagnosis of active TB disease in young children is less than compelling[1], [5], [6], [7]. Indeed, IGRAs have not yet been incorporated into TB control programmes in high-burden developing countries where IGRA sensitivity may be lower, and where healthcare resources and laboratory capacity are most limited[8]. It follows that evidence to guide the use and interpretation of TSTs remains relevant to clinicians and public health programmes in high-burden regions.

The intradermal Mantoux test was adopted as *de facto* standard of care in many developed countries, based on the high rate of false negative results in studies using the percutaneous multi-puncture Tine method during the 1970's and 1980's[9], [10], [11], [12]. For example, Lunn and Johnson reported for the British Thoracic Association that the Tine test was unsuitable for epidemiological use, because of the high proportion of negative results in subjects with a positive Mantoux[11]. Although these findings generated controversy and were contradicted by several studies since 1965, which recommended the Tine test for use in resource-limited settings, multi-puncture TST methods fell into disrepute[2], [13], [14], [15], [16]. This may be unfortunate, since the disposable Tine tool offers potential advantages over the Mantoux method, including rapid application; less wastage; lower unit cost; and lower operator skill level. These potential advantages are counter-balanced by inconsistent delivery of tuberculin by multi-puncture Tine tools, and whereas grading of Tine induration is semi-quantitative, the Mantoux allows more precise measurement[17]. Nevertheless, the advantage of precise measurement of the Mantoux induration is inevitably sacrificed when the test result is categorized as positive or negative, so that it can be interpreted and acted upon by clinicians. Threshold values of 5 mm, 10 mm, and 15 mm have all been used by the American Thoracic Society (ATS), Centers for Disease Control and Prevention (CDC), South African National Tuberculosis Control Programme, and World Health Organization (WHO) for categorical interpretation of the Mantoux result in various TB risk categories and TB prevalence settings[18], [19], [20], [21]. Although these threshold values may have been selected using the best available evidence, it must be acknowledged that the actual values may have little biological meaning. These problems are amplified by the fact that the traditional threshold values are commonly associated with digit preference, which may result in misclassification errors[22].

It is also accepted that both the Mantoux and Tine methods may be subject to factors causing false positive or negative results, including BCG vaccination, NTM exposure, malnutrition, and human immunodeficiency virus (HIV) infection[23]. Several studies have attempted to identify factors associated with TST positivity in order to optimize contact tracing strategies, but the majority of studies have either been small scale, or performed among older children and adults, in developed countries with low TB prevalence, using only the Mantoux method (Table 1) ([24], [25], [26], [27], [28], [29], [30], [31], [32], [33], [34], [35], [36], [37]. Few large studies have included young children, in whom the risk of TB disease is highest, particularly in high prevalence regions of sub-Saharan Africa, and there are no such studies that directly compare intradermal Mantoux and percutaneous multi-puncture Tine methods in the same paediatric population[29], [30], [37]. It was our primary hypothesis that Mantoux and Tine methods would demonstrate moderate agreement in such a study population. Second, we postulated that positive results for both tests would be associated with proximity of exposure to TB contact, and with microbiological and radiological features of TB disease. We present a direct comparison of the intradermal Mantoux and percutaneous multipuncture Tine tests, in which independent risk factors for test positivity are examined in a single study group of young BCG-vaccinated children, in a South African community with very high TB incidence.

**Table 1:** Studies reporting independent (adjusted) risk factors for a positive TST among child contacts of TB cases.

Author	Test	Population	Region	Size (n)	Positive risk factors	Negative risk factors
**Bailey** **[24]**	Mantoux	Adults + children	North America	n = 3528	Case AFB+ smear	Sex female
					Case with CXR cavitation	Age<15 years
					Race other than white	
					Total hours exposed	
**Besser** **[26]**	Mantoux	Children	North America	n = 159	BCG vaccinated	
					Previous TST <12 months ago	
**Carvalho** **[36]**	Mantoux	Adults + children	Europe	n = 360	Household size	
					Case HIV infected	
					Older age	
**Lienhardt** **[29]**	Mantoux	Children	West Africa	n = 285	Family member with positive TST	Duration of cough <10 weeks
					Household proximity to case	
					Household size	
**Lobato** **[34]**	Mantoux	Children	North America	n = 953	Foreign travel	
					Foreign visitor	
					Sex female	
**Rathi** **[31]**	Mantoux	Adults + children	South Asia	n = 385	Older age	
					Sleeping site proximity to case	
					Case AFB+ smear	
					BCG scar	
**Bener** **[35]**	Mantoux	Children	Central Asia	n = 785	Older age	
					Household number of rooms	
					BCG vaccinated	
**Gustafson** **[37]**	Mantoux	Adults + children	West Africa	n = 1980	Older age	Tested in rainy season
					Household case	
					Sleeping site proximity to case	
					Sex female	
					BCG scar	
**Musoke** **[30]**	Mantoux	Children	East Africa	n = 365	Case AFB+ smear	
**Saiman** **[32]**	Mantoux	Children	North America	n = 288	Adult case	Previous negative TST
					Family member with positive TST	
					Foreign birth	
					Foreign travel	
**Froehlich** **[27]**	Mantoux	Children	North America	n = 31926	BCG vaccinated	
					Ethnicity Asian/Hispanic	
					Family member with positive TST	
					Foreign birth	
					Foreign travel	
**Young** **[33]**	Mantoux	Children	North America	n = 584	Birth in Mexico	
					Travel to Mexico	
					Older age	
					BCG vaccinated	

## Methods

This analysis is based on data collected during a BCG vaccine trial in a rural area near Cape Town, South Africa, during 2001–2006 (Clinical Trials identifier: NCT00242047)[38]. A total of 11680 healthy newborns were followed up for a minimum of 2 years (maximum 4.7) after vaccination with Tokyo 172 BCG. The incidence of TB in this region in 2006 was reported as 940 per 100 000 population, and >3000 per 100 000 among children younger than 2 years of age[38], [39], [40]. All children with a TB contact, or symptoms compatible with TB, were identified by community surveillance and the 1869 children who subsequently underwent standardized TB case investigation were eligible for inclusion in this analysis. HIV infection status was determined by rapid antibody test and confirmatory Polymerase Chain Reaction (PCR). Chest radiographs were reviewed by a panel of expert paediatricians for compatibility with a diagnosis of TB. Two consecutive, early morning, paired gastric lavages and induced sputa were obtained for auramine staining and culture of *Mycobacterium tuberculosis* as previously described[41]. The trial was approved by the University of Cape Town Research Ethics Committee (UCTREC 271/2000) and written informed consent was obtained from parents/guardians.

Children underwent simultaneous Tine and Mantoux tuberculin skin tests. The disposable Tine disc (Lederle Laboratories, Philadelphia, USA) was applied percutaneously to the right forearm and 2 units of purified tuberculin protein derivative (PPD) (Statens Serum Institut, Copenhagen, Denmark) were injected intradermally to the left forearm. Due to a temporary stock shortage, some children did not receive a Tine test and therefore paired results were available for 1512 of 1869 children (80.9%). TSTs were read at 48–72 hours and readers were not blinded. Each Tine and Mantoux result was classified both according to a categorical hierarchy, and according to a binary (positive/negative) category, using an approach described previously[12], [13]. The Tine result was ranked in 5 ascending categories described previously: Grade 0 (no indurated papules); Grade 1 (induration of one or more discrete papules); Grade 2 (confluent induration of two or more papules); Grade 3 (confluent plateau induration of all papules); and Grade 4 (confluent blistering)[12], [13], [14], [15]. Similarly, the Mantoux result was ranked in 5 ascending categories (0–4 mm; 5–9 mm; 10–14 mm; 15–19 mm; and ≥20 mm), based upon historical comparisons of Mantoux and Tine tests; traditional threshold values recommended by the ATS, CDC, and WHO; and recent data suggesting a higher cut-off for BCG-vaccinated infants [12], [13], [14], [15], [18], [19], [20]. A strongly positive skin test reaction was used to define the trial end-point (diagnosis of TB disease among very young BCG-vaccinated children in a high prevalence area), as per prevailing WHO and South African national TB programme guidelines[18], [21], [42]. Therefore, the *per protocol* definition of a positive TST result was confluent plateau induration of all Tine papules and/or blistering (Grade 3 or 4 reaction), or Mantoux induration measuring ≥15 mm in the horizontal diameter. Alternative lower threshold values (Tine Grade 2 reaction and Mantoux induration ≥10 mm) and lower hierarchical Mantoux categories were examined in sensitivity analyses. Kappa statistics were generated to examine binary (positive/negative) agreement between TST methods and weighted kappa statistics were calculated to examine categorical hierarchical agreement. The strength of agreement was defined as follows: kappa 0–0.2 = slight; 0.2–0.4 = fair; 0.4–0.6 = moderate; 0.6–0.8 = substantial; and 0.8–1.0 = almost perfect agreement[43].

Continuous data are presented as median and interquartile range (IQR). Categorical data are presented as n (%). Crude associations between positive TST and demographic, TB disease, and TB exposure factors were examined using the Mann-Whitney test for non-parametric continuous data and the McNemar's or chi-squared tests for categorical data. Separate multivariate logistic regression models were built to identify independent risk factors for positive (binary) outcomes for the Mantoux and Tine tests. Manual stepwise nested model selection was used to identify the variables for inclusion in the final logistic model. After adjusting for potential confounding variables, odds ratios (95% confidence intervals) were calculated with positive Mantoux or Tine test as the outcome variable. All statistical analyses were performed using STATA Version 10 (StataCorp, College Station, Texas).

## Results

The study population (n = 1512) included 764 (50.5%) males and 748 (49.5%) females, median age 14.5 months (IQR 13.7 months), and median weight-for-height Z-score 0.05 (IQR 1.79). A history of cough >2 weeks was reported in 645 (42.7%) and fever was reported in 583 children (38.6%). The median household size was 6 members. A TB contact was reported outside of the household in 298 children (19.7%), inside the household, but excluding the child's mother, in 551 children (36.4%), and a maternal TB contact was reported in 144 children (9.5%). HIV ELISA was positive in 47 children, of whom 30 (2.0%) were confirmed HIV infected. The chest radiograph was suggestive of TB disease in 298 (19.7%) and *M. tuberculosis* was cultured in 173 children (11.4%).

**Figure 1:**
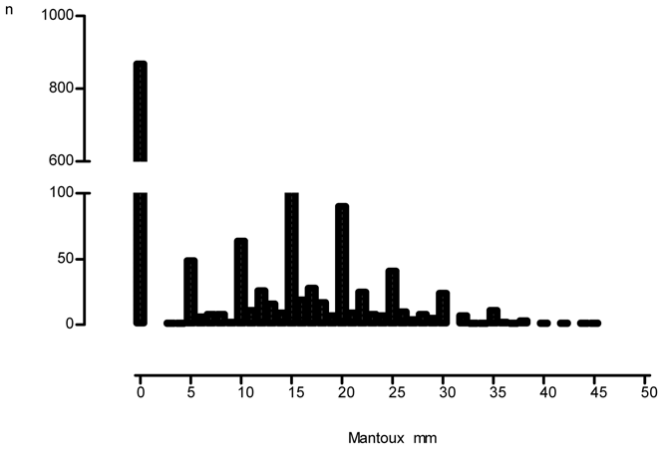
Histogram showing distribution of Mantoux induration diameter (n = 1512) with evidence of reader digit preference at the 5 mm, 10 mm, 15 mm, and 20 mm values.

The Mantoux test was positive at the 15 mm threshold in 430 of 1512 children (28.4%) and the Tine test was positive at the Grade 3 threshold in 496 children (32.8%), (p<0.0001). The Mantoux detected 77% of 559 children with a positive test result by either TST method (37.0%), compared to the Tine which detected 89% (p<0.001). Conversely, 59% of children had a Mantoux reaction <5 mm diameter, compared to 41% of children with a Grade 0 Tine reaction (p<0.001). The distribution plot of the Mantoux induration diameter suggests that reader digit preference occurred at the 5 mm, 10 mm, 15 mm, and 20 mm threshold values (Figure 1).

Observed agreement for comparison of binary positive/negative TST outcomes was 87.3%, with unweighted kappa = 0.70 (Table 2).

**Table 2:** Agreement between Mantoux and Tine tests for binary comparisons (positive/negative test) among 1512 children (kappa = 0.70).

Test	Tine Positive	Tine Negative	Total
**Mantoux Positive**	n = 367 (24.3%)	n = 63 (4.2%)	**n = 430 (28.4%)**
**Mantoux Negative**	n = 129 (8.5%)	n = 953 (63.0%)	**n = 1082 (71.6%)**
**Total**	**n = 496 (32.8%)**	**n = 1016 (67.2%)**	**n = 1512 (100%)**

Observed agreement for hierarchical TST outcomes was 85%, with weighted kappa = 0.66 (Table 3).

**Table 3:** Agreement between Mantoux and Tine tests for ordered categorical comparisons (Mantoux <5 mm, ≥5 to 10 mm, ≥10 to 15 mm, ≥15 to 20 mm, ≥20 mm, and Tine Grades 0–4), among n = 1512 children (weighted kappa = 0.66).

Test	Tine (Grade)	0	1	2	3	4	Total
Mantoux (mm)							
**0–4**		n = 600 (39.7%)	n = 169 (11.2%)	n = 81 (5.4%)	n = 27 (1.8%)	n = 15 (1.0%)	**n = 892 (59.0%)**
**5–9**		n = 12 (0.8%)	n = 11 (0.7%)	n = 28 (1.9%)	n = 11 (0.7%)	n = 6 (0.4%)	**n = 68 (4.5%)**
**10–14**		n = 2 (0.1%)	n = 12 (0.8%)	n = 38 (2.5%)	n = 32 (2.1%)	n = 38 (2.5%)	**n = 122 (8.1%)**
**15–20**		n = 5 (0.3%)	n = 12 (0.8%)	n = 27 (1.8%)	n = 40 (2.7%)	n = 89 (5.9%)	**n = 173 (11.4%)**
**≥20**		n = 2 (0.1%)	n = 5 (0.3%)	n = 12 (0.8%)	n = 30 (2.0%)	n = 208 (13.8%)	**n = 257 (17.0%)**
**Total**		**n = 621 (41.1%)**	**n = 209 (13.82%)**	**n = 186 (12.3%)**	**n = 140 (9.26%)**	**n = 356 (23.55%)**	**n = 1512 (100%)**

### Sub-Group Analysis

Among 173 children culture-positive for *Mycobacterium tuberculosis,* the TST was positive by at least one method in 103 cases (59.5%). The Mantoux was positive in 85 children (49.1%) and the Tine in 95 children (54.9%), p<0.0001, with 85.0% observed agreement and kappa = 0.70. However, observed agreement for hierarchical categories was 56.7% (kappa = 0.42), with the predominant source of disagreement arising from cases in which the Tine result lay in a higher category than the Mantoux result (data not shown). Fifty-nine children (34.1%) showed a Mantoux reaction <5 mm diameter, compared to 41 children (23.7%) with a Grade 0 Tine reaction (p<0.0001).

### Univariate Analysis

The odds of a positive Mantoux test were increased by demographic factors, including mixed ancestry and increasing age; TB disease factors, including suggestive chest radiograph, culture of *M. tuberculosis*, previous TB treatment; other factors, such as previous anthelmintic treatment; and TB exposure factors, including a household TB contact (other than mother), as well as a maternal TB contact. Wheezing and HIV infection were both associated with a negative Mantoux test. The factors of mixed ancestry, female gender, increasing age, suggestive chest radiograph, culture of *M. tuberculosis,* previous TB treatment, household TB contact, and maternal TB contact, were risk factors for a positive Tine test. HIV infection, fever, and increasing sibling numbers, were also associated with a negative Tine test (Table 4).

**Table 4:** Risk factors (odds ratios and 95% confidence intervals, CI) associated with a positive Mantoux or Tine test (univariate analysis) among n = 1512 children investigated for TB.

	Mantoux OR (95% CI)	Tine OR (95%CI)
**Demographic factors**		
Mixed ancestry	1.64 (1.16–2.30))	1.74 (1.25–2.41)
Gender female	1.25 (0.99–1.56)	1.68 (1.35–2.09)
Age (months)	1.01 (1.00–1.03)	0.99 (0.98–1.00)
Weight for height z-score	0.94 (0.87–1.02)	0.98 (0.92–1.05)
Height for age z-score	1.05 (0.98–1.13)	0.94 (0.87–1.02)
Weight for age z-score	0.99 (0.92–1.09)	0.95 (0.89–1.02)
**TB disease factors**		
Suggestive chest radiograph	2.28 (1.76–2.95)	1.82 (1.39–2.39)
Previous TB treatment	2.15 (1.69–2.71)	1.65 (1.29–2.11)
Culture of *M.Tuberculosis*	2.49 (1.78–3.48)	2.83 (1.99–4.04)
AFB smear positive	0.31 (0.03–1.32)	4.11 (0.59–45.5)
Wheezing	0.65 (0.52–0.82)	0.85 (0.68–1.07)
Cough >2 weeks	0.19 (0.68–1.09)	0.92 (0.75–1.14)
Fever	0.81 (0.64–1.01)	0.65 (0.51–0.82)
Night sweats	0.85 (0.68–1.08)	0.98 (0.79–1.21)
**Other factors**		
Previous anthelmintic treatment	1.36 (1.07–1.73)	0.97 (0.76–1.24)
HIV infection	0.08 (0.00–0.51)	0.15 (0.02–0.62)
**TB exposure factors**		
**Overcrowding**		
Number of siblings	0.96 (0.87–1.05)	0.93 (0.86–1.00)
Number of household members	1.02 (0.98–1.06)	1.03 (0.99–1.06)
**TB contact**		
None	1.00	1.00
Outside household	0.85 (0.64–1.12)	0.91 (0.68–1.19)
Inside household (excluding mother)	1.5 (1.2–1.87)	1.45 (1.15–1.82)
Mother	2.94 (2.08–4.13)	3.3 (2.29–4.77)

### Multivariate Analysis

In the multivariate analysis, the adjusted odds of a positive Mantoux test were increased by suggestive chest radiograph, culture of *M. tuberculosis*, previous anthelmintic treatment, and three categories of TB contact (outside the household, inside the household excluding mother, and maternal TB contact, which demonstrated the strongest association). Weight for height Z-score between -1 and zero, compared to a more severe score, was associated with a negative Mantoux. Suggestive chest radiograph, culture of *M. tuberculosis*, the same three categories of TB contact, and previous TB treatment, were independent risk factors for a positive Tine test. Weight for height Z-score between -1 and zero was also associated with a negative Tine test, as was the presence of fever, and HIV infection (Table 5).

**Table 5:** Independent risk factors (odds ratios, OR, and 95% confidence intervals, CI) associated with a positive Mantoux or Tine test among n = 1512 children investigated for TB (multivariate logistic regression model).

	Mantoux test positive		Tine test positive	
	OR 95%CI	p	OR 95%CI	p
Weight for height Z-score (−1,0)	0.71 (0.53–0.95)	0.02	0.64 (0.48–0.86)	<0.0001
Suggestive chest radiograph	2.31 (1.71–3.11)	<0.0001	2.06 (1.51–2.81)	<0.0001
Previous TB treatment	------	------	1.56 (1.17–2.09)	<0.0001
Culture of *M. tuberculosis*	2.19 (1.49–3.24)	<0.0001	2.47 (1.65–3.69)	<0.0001
Fever	------	------	0.70 (0.53–0.93)	<0.0001
Previous anthelmintic treatment	1.41 (1.06–1.87)	0.02	----	-----
HIV infection	------	------	0.09 (0.01–0.74)	0.02
TB contact outside household	1.86 (1.26–2.72)	<0.0001	1.98 (1.37–2.88)	<0.0001
TB contact inside household (excluding mother)	2.53 (1.81–3.53)	<0.0001	2.28 (1.64–3.18)	<0.0001
TB contact mother	5.43 (3.46–8.53)	<0.0001	5.47 (3.39–8.81)	<0.0001

### Sensitivity Analysis

The effect of lowering the TST threshold values was compared to the *per protocol* results above. Lowering the threshold for a positive test result to 10 mm Mantoux and Grade 2 Tine yielded similar observed agreement (86.4%) and unweighted kappa = 0.72. Lowering the Mantoux hierarchy ranking by one category, that is Mantoux 0 mm; 1–4 mm; 5–9 mm; 10–14 mm; and ≥15 mm, compared to Tine Grades 0–4, also demonstrated similar observed agreement (84.4%) and weighted kappa = 0.64. In the multivariate model, lowering the threshold for positive Mantoux to 10 mm yielded a similar set of variables associated with a positive test result, with the exception that previous anthelmintic treatment (risk factor) exited the model and previous TB treatment (risk factor) and wheezing (protective factor) entered the model.

## Discussion

We have shown in this large study of young children with suspected tuberculosis that there is substantial agreement between the Mantoux and Tine methods, both for the binary comparison and for hierarchical categories of increasing skin test reactivity. This level of agreement occurred in the presence of evidence of reader digit preference for Mantoux values occurring at 5 mm threshold intervals and similar findings were obtained in sensitivity analyses using alternative threshold values for a positive test. Further, even though a relatively high positive test threshold was defined, 37% of all children, and 59% of culture-positive children, had a positive TST result by at least one method. In contrast to several previous studies, the proportion of children with a positive Tine was significantly greater than the proportion with a positive Mantoux, and the Tine had a lower rate of minimally reactive/unreactive tests than the Mantoux method[9], [10], [11], [12]. These findings also held true for the sub-group of children with a positive culture of *M. tuberculosis*.

There are no large studies that describe direct comparison of Mantoux and Tine results in the last two decades, either among children or adults, although earlier adult studies reported high rates of false-negative Tine results among participants who were Mantoux positive[9], [10], [11], [12]. For example, the influential study by Lunn and Johnson among 307 students showed a Mantoux positive rate of 59%, compared to a Tine positive rate of 3.9%, with 40% of all readings Tine negative and Mantoux positive[11]. Clearly, categorical agreement and positive test rate do not measure the accuracy of either test since, in the absence of a gold standard for latent tuberculosis infection, a positive TST may reflect BCG or NTM exposure. However, our findings among *M. tuberculosis* culture-positive children imply that the Tine test may have equivalent or higher detection rates for mycobacterial antigen exposure among young BCG-vaccinated children in this population. Therefore, given the potential savings in consumables, wastage, and operator time, as well as the lesser skill level needed, it would be reasonable to recommend the multi-puncture Tine as a screening tool for childhood TB in developing regions with limited funds, equipment, and a shortage of health care personnel.

We have shown in a multivariate analysis that the odds of a positive Mantoux among children with suspected TB were increased if the chest radiograph were suggestive of pulmonary TB disease, and if *M. tuberculosis* were cultured from gastric lavage fluid or from induced sputum. This would be expected among children with prior TB infection that progressed to active disease, and supports the inclusion of the Mantoux test result in the diagnostic decision-making pathway for childhood TB, even in high TB prevalence areas. All three categories of TB contact were independent risk factors for a positive Mantoux, with the magnitude of the association increasing in proportion to the proximity of contact, and with maternal contact being the strongest risk factor. These findings are consistent with increased household exposure to family TB contacts among younger children who spend the majority of time with their mothers. These data also emphasize the importance of contact tracing and LTBI prophylaxis for household contacts of smear positive adults in national TB control programmes. Many of the risk factors for a positive Mantoux test were common to the Tine test, including a suggestive chest radiograph, culture of *M. tuberculosis*, and the same three categories of TB contact.

The presence of fever and HIV infection were both independently associated with a negative Tine test, although these variables did not enter the final Mantoux regression model. HIV infected children might be expected to have less skin test reactivity on the basis of immune-suppression. However, our finding of a negative association between HIV infection and the Tine test result is in contrast to studies in the USA and Uganda[30], [32]. Fever is a feature of TB disease, but the fact that fever was associated with a negative Tine test might be explained if fever in the children being investigated was primarily on the basis of infections other than TB, or if children with fever were more severely ill. We have shown that a weight-for-height Z-score between -1 and zero was protective against positive Mantoux and Tine tests, compared to more severe Z-scores, although others have reported no independent relationship between TST result and nutritional status[29], [30]. It is possible that in our study, more severe weight-for-height Z-scores might have been associated with a positive TST in the setting of active TB disease.

Several demographic factors associated with positive Mantoux and Tine tests in the univariate analysis, such as ethnicity, gender, and age, were excluded from the multivariate regression model as dependant co-variables, suggesting that TST positivity is primarily related to TB exposure rather than demography. Interpretation of the literature is complicated by the fact that studies of risk factors for TST positivity have been conducted in populations with very different demographic, public health, and clinical characteristics[24], [25], [27], [30], [32], [33], [35], [36], [37]. For example, in our study there were no independent associations between TST result and measures of overcrowding, even though households were relatively large, similar to findings in the UAE and Uganda, but in contrast to a study in Brazil[30], [35], [36].

This study has several notable limitations. All children had been BCG-vaccinated and lived in the same high TB prevalence area, so we were not able to examine potential associations between these factors and TST positivity. Similarly, the findings apply only to young children under the age of five years, who form the age group with the greatest relative burden of TB disease. It is also not possible to assess the performance of either TST among those children with a clinical diagnosis of TB from whom *M. tuberculosis* was not cultured, since the TST result formed part of this diagnostic algorithm[38].

The *per protocol* definition of TST positivity was designed to aid diagnosis of TB disease among young BCG-vaccinated children, in accordance with the prevailing national and international guidelines[18], [21], [42]. This threshold level is higher than that currently advocated by some authorities, although it has recently been suggested that the optimal threshold for a positive Mantoux in BCG-vaccinated infants may be even greater[44], [45], [46]. It might also be argued that use of a threshold to dichotomize positive and negative tests ignores the value of precise quantitative measurements offered by the Mantoux method, resulting in bias towards the semi-quantitative Tine test. However, we suggest that this approach mirrors clinical practice, in that clinical management decisions are likely to be made on the basis of a positive or negative TST result. It is true that much of the inherent complexity of precise measurement is lost when the Mantoux test result is categorized as positive or negative for clinical purposes. Threshold values of 5 mm, 10 mm, and 15 mm have all been used by the ATS, CDC, South African National Tuberculosis Control Programme, and WHO for categorical interpretation of the Mantoux result in various TB risk categories and TB prevalence settings, although it must be acknowledged that the actual values may have little biological meaning[18], [19], [20], [21]. Inaccuracy of the Mantoux method may be falsely exaggerated by the fact that the traditional threshold values are commonly associated with digit preference, as we have demonstrated, which may result in misclassification errors that are difficult to correct for individual readings[22], [47]. However, although digit preference tends to undercount both those values immediately above and immediately below the preferred threshold values, the practical outcome of digit preference is usually misclassification into higher, rather than lower, Mantoux categories (eg. ≥5 mm; ≥10 mm; and ≥15 mm)[22]. Therefore, although it is possible that the substantial agreement between categorical Mantoux and categorical Tine results is influenced in some way by digit preference, this factor does not explain why the Tine test demonstrated a significantly higher positive test rate than the Mantoux, since the opposite observation would have been expected.

Since we lack a diagnostic gold standard for latent tuberculosis infection, we are unable to measure the accuracy of either TST method for this purpose, and we acknowledge that the higher positive Tine rate might reflect BCG or NTM exposure. The limited sensitivity of the Mantoux among culture-positive children (49%) may be due, in part, to operator error in the intradermal administration of tuberculin, as well as the suppressive effect of TB disease on cell-mediated immunity[4]. Therefore, although we recommend the Tine as a suitable screening tool in developing regions, further comparative studies would be needed before the Tine method could be suggested for definitive diagnosis of childhood TB disease. These recommendations might appear retrogressive, given the current research interest in IGRAs[4]. However, the effect of limited healthcare resources on the reality of TB control in high-burden countries is illustrated by the fact that, until recently, even Mantoux testing has not been available in some parts of East Africa[48]. It is likely to be several years before the IGRA might replace tuberculin skin testing in regions where childhood TB is most prevalent[1]

In summary, we have shown in a large study of BCG-vaccinated infants and children who were investigated for TB in a high prevalence area that the Tine method has a higher positive test rate than the Mantoux, both overall and among children who were culture-positive for *M. tuberculosis*, with substantial agreement between TSTs for binary and hierarchical comparisons. This level of agreement occurred in the presence of evidence of reader digit preference in recording the Mantoux diameter. Multivariate models showed that independent risk factors for both Tine and Mantoux tests include TB disease factors, such as a suggestive chest radiograph or culture of *M. tuberculosis*, and TB exposure factors, with maternal TB contact most strongly associated with the risk of a positive TST. Ethnicity, age, gender, and measures of overcrowding were not independent predictors of the TST result. In contrast to previous adult studies, these data suggest that the Tine may be a reasonable alternative to the Mantoux method as a screening tool for childhood TB in resource-limited, high-burden areas, or in settings where convenience and ease of use are major considerations[9], [10], [11], [12].

## References

[1] Shingadia D, Novelli V (2008). The tuberculin skin test: a hundred, not out?. Arch Dis Child.

[2] Rosenthal SR, Nikurs L, Yordy E, Hoder B, Thorne M (1965). The tuberculin tine test compared to the Mantoux test with 5, 10, and 100 tuberculin units.. Pediatrics.

[3] Hesseling AC, Gie RP (2007). Scoring systems for the diagnosis of childhood tuberculosis: are we making progress?. Int J Tuberc Lung Dis.

[4] Menzies D, Pai M, Comstock G (2007). Meta-analysis: new tests for the diagnosis of latent tuberculosis infection: areas of uncertainty and recommendations for research.. Ann Intern Med.

[5] Mazurek GH, Villarino ME (2003). Guidelines for using the QuantiFERON-TB test for diagnosing latent Mycobacterium tuberculosis infection. Centers for Disease Control and Prevention.. MMWR Recomm Rep.

[6] Nicol MP, Davies MA, Wood K, Hatherill M, Workman L (2009). Comparison of T-SPOT.TB assay and tuberculin skin test for the evaluation of young children at high risk for tuberculosis in a community setting.. Pediatrics.

[7] Bianchi L, Galli L, Moriondo M, Veneruso G, Becciolini L (2009). Interferon-gamma Release Assay Improves the Diagnosis of Tuberculosis in Children.. Pediatr Infect Dis J.

[8] Dheda K, Smit RZ, Badri M, Pai M (2009). T-cell interferon-gamma release assays for the rapid immunodiagnosis of tuberculosis: clinical utility in high-burden vs. low-burden settings.. Curr Opin Pulm Med.

[9] Browder AA, Griffon AL (1972). Tuberculin tine tests on medical wards.. Am Rev Respir Dis.

[10] Hansen JP, Falconer JA, Gallis HA, Hamilton JD (1982). Inadequate sensitivity of tuberculin tine test for screening employee populations.. J Occup Med.

[11] Johnson A, Lunn JA (1978). Comparison of the time and Mantoux tuberculin tests.. Br Med J.

[12] Welke H, Irsigler GB, Kleeberg HH (1976). The diagnostic value of the tine and Mantoux tests in a general hospital.. S Afr Med J.

[13] Freiman I, Hartman E, Abkiewicz C (1976). A comparative study of tuberculin tine and Mantoux tests.. S Afr Med J.

[14] Rudd RM, Gellert AR, Venning M (1982). Comparison of Mantoux, tine, and ‘Imotest’ tuberculin tests.. Lancet.

[15] Sinclair DJ, Johnston RN (1979). Assessment of tine tuberculin test.. Br Med J.

[16] Snell NJ (1979). A comparison of Mantoux and tuberculin Tine testing in a chest unit.. Tubercle.

[17] Lunn JA (1980). Reason for variable response to tine test.. Br Med J.

[18] WHO (2003). Treatment of tuberculosis: Guidelines for national programmes.. WHO/CDS/TB/2003313 3rd Edition: Page 63.

[19] American Thoracic Society (2000). Targeted tuberculin testing and treatment of latent tuberculosis infection.. MMWR Recomm Rep.

[20] Joint Statement of the American Thoracic Society (ATS) and the Centers for Disease Control and Prevention (CDC) (2000). Targeted tuberculin testing and treatment of latent tuberculosis infection.. Am J Respir Crit Care Med.

[21] DOH (2004). The South African National Tuberculosis Control Programme Practical Guidelines 2004..

[22] Eilers PH, Borgdorff MW (2004). Modeling and correction of digit preference in tuberculin surveys.. Int J Tuberc Lung Dis.

[23] Black GF, Dockrell HM, Crampin AC, Floyd S, Weir RE (2001). Patterns and implications of naturally acquired immune responses to environmental and tuberculous mycobacterial antigens in northern Malawi.. J Infect Dis.

[24] Bailey WC, Gerald LB, Kimerling ME, Redden D, Brook N (2002). Predictive model to identify positive tuberculosis skin test results during contact investigations.. JAMA.

[25] Ozuah PO, Ozuah TP, Stein RE, Burton W, Mulvihill M (2001). Evaluation of a risk assessment questionnaire used to target tuberculin skin testing in children.. JAMA.

[26] Besser RE, Pakiz B, Schulte JM, Alvarado S, Zell ER (2001). Risk factors for positive mantoux tuberculin skin tests in children in San Diego, California: evidence for boosting and possible foodborne transmission.. Pediatrics.

[27] Froehlich H, Ackerson LM, Morozumi PA (2001). Targeted testing of children for tuberculosis: validation of a risk assessment questionnaire.. Pediatrics.

[28] Joos TJ, Miller WC, Murdoch DM (2006). Tuberculin reactivity in bacille Calmette-Guerin vaccinated populations: a compilation of international data.. Int J Tuberc Lung Dis.

[29] Lienhardt C, Sillah J, Fielding K, Donkor S, Manneh K (2003). Risk factors for tuberculosis infection in children in contact with infectious tuberculosis cases in the Gambia, West Africa.. Pediatrics.

[30] Mudido PM, Guwatudde D, Nakakeeto MK, Bukenya GB, Nsamba D (1999). The effect of bacille Calmette-Guerin vaccination at birth on tuberculin skin test reactivity in Ugandan children.. Int J Tuberc Lung Dis.

[31] Rathi SK, Akhtar S, Rahbar MH, Azam SI (2002). Prevalence and risk factors associated with tuberculin skin test positivity among household contacts of smear-positive pulnionary tuberculosis cases in Umerkot, Pakistan.. Int J Tuberc Lung Dis.

[32] Saiman L, San Gabriel P, Schulte J, Vargas MP, Kenyon T (2001). Risk factors for latent tuberculosis infection among children in New York City.. Pediatrics.

[33] Young J, O'Connor ME (2005). Risk factors associated with latent tuberculosis infection in Mexican American children.. Pediatrics.

[34] Lobato MN, Hopewell PC (1998). Mycobacterium tuberculosis infection after travel to or contact with visitors from countries with a high prevalence of tuberculosis.. Am J Respir Crit Care Med.

[35] Bener A, Uduman S, Bin-Othman SA (1996). Factors associated with tuberculin reactivity among children in United Arab Emirates.. Respir Med.

[36] Carvalho AC, DeRiemer K, Nunes ZB, Martins M, Comelli M (2001). Transmission of Mycobacterium tuberculosis to contacts of HIV-infected tuberculosis patients.. Am J Respir Crit Care Med.

[37] Gustafson P, Lisse I, Gomes V, Vieira CS, Lienhardt C (2007). Risk factors for positive tuberculin skin test in Guinea-Bissau.. Epidemiology.

[38] Hawkridge A, Hatherill M, Little F, Goetz MA, Barker L (2008). Efficacy of percutaneous versus intradermal BCG in the prevention of tuberculosis in South African infants: randomised trial.. BMJ.

[39] Groenewald P BOROWorcester (2004). Boland Overberg Region Annual Health Status Report..

[40] WHO (2008). Global tuberculosis control. Country profile. South Africa.. http://www.who.int/globalatlas.

[41] Hatherill M, Hawkridge T, Zar HJ, Whitelaw A, Tameris M (2009). Induced sputum or gastric lavage for community-based diagnosis of childhood pulmonary tuberculosis?. Arch Dis Child.

[42] DOH (2000). The South African National Tuberculosis Control Programme Practical Guidelines 2000..

[43] McGinn T, Wyer PC, Newman TB, Keitz S, Leipzig R (2004). Tips for learners of evidence-based medicine: 3. Measures of observer variability (kappa statistic).. CMAJ.

[44] Chan PC, Chang LY, Wu YC, Lu CY, Kuo HS (2008). Age-specific cut-offs for the tuberculin skin test to detect latent tuberculosis in BCG-vaccinated children.. Int J Tuberc Lung Dis.

[45] CDC (2005). Guidelines for the investigation of contacts of persons with infectious tuberculosis. Recommendations from the National Tuberculosis Controllers Association and CDC.. MMWR Recomm Rep.

[46] DOH (2008). The South African National Tuberculosis Control Programme Practical Guidelines 2008..

[47] Davies GR, Fine PE, Vynnycky E (2006). Mixture analysis of tuberculin survey data from northern Malawi and critique of the method.. Int J Tuberc Lung Dis.

[48] Braitstein P, Nyandiko W, Vreeman R, Wools-Kaloustian K, Sang E (2009). The Clinical Burden of Tuberculosis Among Human Immunodeficiency Virus-Infected Children in Western Kenya and the Impact of Combination Antiretroviral Treatment.. Pediatr Infect Dis J.

